# Histopathological Evaluation of Somatostatin Receptor 2 Expression in Myocarditis—Rationale for the Diagnostic Use of Somatostatin Receptor Imaging

**DOI:** 10.3390/diagnostics14212374

**Published:** 2024-10-24

**Authors:** Christian L. Polte, Kittichate Visuttijai, Kristina Vukusic, Joakim Sandstedt, Mikael Sandstedt, Emanuele Bobbio, Marie Björkenstam, Kristjan Karason, Niklas Bergh, Entela Bollano, Anders Oldfors

**Affiliations:** 1 Department of Clinical Physiology, Sahlgrenska University Hospital, 41345 Gothenburg, Sweden; 2 Department of Radiology, Sahlgrenska University Hospital, 41345 Gothenburg, Sweden; 3 Institute of Medicine, Sahlgrenska Academy, University of Gothenburg, 41390 Gothenburg, Sweden; 4 Department of Clinical Pathology, Sahlgrenska University Hospital, 41345 Gothenburg, Sweden; 5 Institute of Biomedicine, Sahlgrenska Academy, University of Gothenburg, 41390 Gothenburg, Sweden; 6 Department of Clinical Chemistry, Sahlgrenska University Hospital, 41345 Gothenburg, Sweden; 7 Department of Cardiology, Sahlgrenska University Hospital, 41345 Gothenburg, Sweden; 8 Department of Transplantation, Sahlgrenska University Hospital, 41345 Gothenburg, Sweden

**Keywords:** somatostatin receptor, histopathology, myocarditis, giant-cell myocarditis, cardiac sarcoidosis, imaging, somatostatin receptor imaging, positron emission tomography

## Abstract

Background/Objectives: Myocarditis is an inflammatory disease of the myocardium and remains to this day a challenging diagnosis. A promising novel imaging method uses the expression of somatostatin receptors (SSTRs) on inflammatory cells to visualize myocardial inflammation. However, little is known about the histopathological correlate of SSTR imaging in different forms of myocarditis. Methods: In the present retrospective histopathological study, we systematically analysed the expression of SSTR subtype 2 (SSTR2) on inflammatory cells of 33 patients with biopsy- or explant-proven myocarditis (lymphocytic myocarditis (*n* = 5), giant-cell myocarditis (*n* = 11), and cardiac sarcoidosis (*n* = 17)), and in eight controls (multi-organ donors) without signs of myocardial inflammation and/or scars. Results: In all patients, immunohistochemical staining for SSTR2 was positive in areas with CD68-positive macrophages and multinucleated giant cells. Staining for SSTR2 was most prominent in the presence of multinucleated giant cells. The colocalization of both SSTR2 and CD68 on the same cell could be confirmed using immunofluorescence microscopy. Western blotting confirmed the upregulated expression of SSTR2 in cases of granulomatous inflammation (sarcoidosis) of the skeletal and heart muscle, in comparison with controls. Conclusions: In conclusion, our findings demonstrate the expression of SSTR2 on the protein level on CD68-positive macrophages and multinucleated giant cells in various forms of myocarditis, which provides a clear rationale for the diagnostic use of SSTR imaging in this patient group.

## 1. Introduction

Myocarditis is a common inflammatory disease of the myocardium, and remains to this day a challenging diagnosis, due to its heterogeneity of clinical presentation and broad spectrum of underlying aetiologies [1]. Most frequently, myocarditis is caused by viral infections resulting in a so-called lymphocytic myocarditis (LM), which typically has a mild course and resolves spontaneously [2]. In contrast, giant-cell myocarditis (GCM) and cardiac sarcoidosis (CS) are both rare and potentially fatal forms of myocarditis [3,4,5,6]. The reference standard for establishing the diagnosis of myocarditis is still an endomyocardial biopsy, although the method faces several limitations, mainly due to its invasive nature, infrequent clinical use, and overall low sensitivity [7,8]. In clinical practice, non-invasive imaging techniques like cardiovascular magnetic resonance and positron emission tomography/computed tomography (PET/CT) have become an integral cornerstone in the diagnostic work-up of patients with clinically suspected myocarditis [9]. However, even these advanced imaging techniques have their limitations.

Somatostatin receptor (SSTR) PET/CT is a novel promising molecular imaging technique that can potentially visualize inflammatory cell infiltration like a biopsy, as SSTRs are not only overexpressed on the surface of most neuroendocrine tumours but also on the surface of activated monocytes, macrophages, and lymphocytes (specifically SSTR subtype 2 (SSTR2) and subtype 3 (SSTR3)), which infiltrate the inflamed myocardium [10,11]. The feasibility of this method in patients with myocarditis has previously been shown in small studies and case reports, including a few cases with histopathological correlations [11,12,13,14,15,16,17,18,19,20,21,22,23]. However, no study has so far been undertaken to systematically characterize the histopathological correlate for SSTR imaging in a larger number of patients with different forms of myocarditis.

Accordingly, the aim of the present study was to obtain additional histopathologic evidence for the expression of SSTR2 on inflammatory cells in patients with biopsy- or explant-proven myocarditis of varying underlying aetiology, which in turn provides a rationale for using SSTR imaging as a diagnostic tool in this patient group.

## 2. Materials and Methods

### 2.1. Study Population and Diagnostic Criteria

This retrospective histopathological study comprised a total of 33 patients with LM (*n* = 5), GCM (*n* = 11), and CS (*n* = 17). Control tissue without signs of myocardial inflammation and/or scar was obtained from eight multi-organ donors, which were not suitable for heart transplantation, but were instead used for research purposes as consented by the donor via the organ donor register or next of kin. Furthermore, tissue samples from patients with granulomatous inflammation (sarcoidosis) of the skeletal (*n* = 1) and heart muscle (*n* = 1) as well as control tissues (cerebellum (*n* = 2), healthy skeletal (*n* = 3) and cardiac muscle (*n* = 2)) were used for Western blot analyses. All patients were identified and randomly selected from our institutional biopsy/explant register, and none of the patients had undergone SSTR imaging. Basic clinical information was obtained with the help of medical records. Patients were eligible for inclusion if their biopsies/explants were of good quality and met the state-of-the-art histopathological diagnostic criteria for LM (Dallas criteria plus immunohistochemical (IHC) staining), GCM (presence of widespread inflammatory cell infiltrate with multinucleated giant cells in association with myocyte damage), or CS (presence of at least one non-necrotizing epithelioid cell granuloma, with or without foci of lymphocytic myocarditis, as well as the absence of both significant myocardial necrosis and notable tissue eosinophilia) [1,24,25]. All analyses were performed by experienced lab personnel and the interpretation was executed by a cardiovascular pathology expert in a blinded manner.

The study was conducted according to the Declaration of Helsinki and approved by the Swedish Ethical Review Authority.

### 2.2. Histological, Immunohistochemical, and Immunofluorescene Analysis

Formalin-fixed and paraffin-embedded biopsies were cut into 3 µm thick sections and dried on a heat plate at 60 °C for one hour. For the histopathological analyses, the tissue sections were stained with Hematoxylin and Eosin, according to established protocols [26]. The sections were pretreated with an antigen-retrieval process using EnVision FLEX Target Retrieval Solution and High pH in PT Link (Agilent Dako, Santa Clara, CA, USA) before IHC and immunofluorescence (IF) analyses. The sections were processed in a Dako Autostainer (Agilent Dako, Santa Clara, CA, USA) for IHC and IF. For IHC, the Dako EnVision FLEX High pH kit (Agilent Dako, Santa Clara, CA, USA) was used. The sections were blocked using 10% normal goat serum for IF. Primary antibodies used for IHC and IF were anti-SSTR2 (ab134152, Abcam, Cambridge, UK), and anti-CD68 (IR609, Agilent Dako, Santa Clara, CA, USA). The secondary antibodies used for the IF analysis were goat anti-mouse IgG, Alexa Fluor™ 647 (A-21235, Thermo Scientific, Waltham, MA, USA), and goat anti-rabbit IgG, Alexa Fluor™ 488 (A-11008, Thermo Scientific, Waltham, MA, USA). The IHC staining intensity of inflammatory cells for SSTR2 was visually graded as follows: prominent (+++), moderate (++), light (+), or absent (−). Illustrative examples of the applied grading technique can be found in Figure S1.

### 2.3. Western Blot Analysis

Western blots were performed on protein extracted from fresh-frozen human cerebellar cortex (positive control), skeletal muscle, and cardiac muscle biopsy specimens from controls and patients with known granulomatous inflammation (sarcoidosis) of the skeletal and cardiac muscle. Protein extraction was performed using an SDS-urea buffer, and 25 μg of extracted protein was loaded per well on NuPAGE 4–12% Bis-Tris gels (Thermo Scientific, Waltham, MA, USA), followed by transfer to polyvinylidene fluoride membranes. The membranes were incubated with primary antibodies overnight at 4 °C and visualized with horseradish peroxidase-conjugated secondary antibodies and SuperSignal West Femto substrate (Thermo Scientific, Waltham, MA, USA). An enhanced chemiluminescent detection Western blotting system (Fujifilm LAS-4000 system, Tokyo, Japan) was used for detection. The primary antibodies used in the analyses were anti-SSTR2 (dilution 1:500, ab134152, Abcam, Cambridge, UK) and anti-GAPDH (dilution 1:500, sc-47724, Santa Cruz Biotechnology, Dallas, TX, USA), as a loading control.

### 2.4. Statistical Analysis

Continuous variables are expressed as median (interquartile range (IQR)), whereas categorical variables are given in numbers and percentages. For continuous variables, significant differences between the groups were assessed using a one-way ANOVA test to determine the overall *p*-value. Rates and proportions were compared using a chi-square test or Fisher’s exact test. Overall, a two-sided *p*-value of <0.05 was considered as significant. Statistical analysis was performed using IBM SPSS Statistics 29 (IBM Corporation, Somers, New York, NY, USA) 

## 3. Results

### 3.1. Patient and Control Characteristics

The median (IQR) age of patients with LM, GCM, and CS was 29 (25–67), 54 (51–67), and 58 (52–62) years, respectively. A female predominance was observed in LM (*n* = 3 (60%)) and GCM (*n* = 6 (55%)), whereas a male predominance was present in CS (*n* = 10 (59%)). More detailed information concerning the demographic and clinical characteristics of the myocarditis patients can be found in Table 1. A recent infection was observed in four patients (80%) with LM, and extracardiac sarcoidosis was present in seven patients (41%) with CS.

Controls had a median (IQR) age of 47 years (29–63) and were mainly of female gender (*n* = 5 (63%)). More detailed information concerning the basic characteristics and clinical background of each individual control can be found in Table 2.

### 3.2. Histological, Immunohistochemical, and Immunofluorescence Characteristics

**Figure 1:**
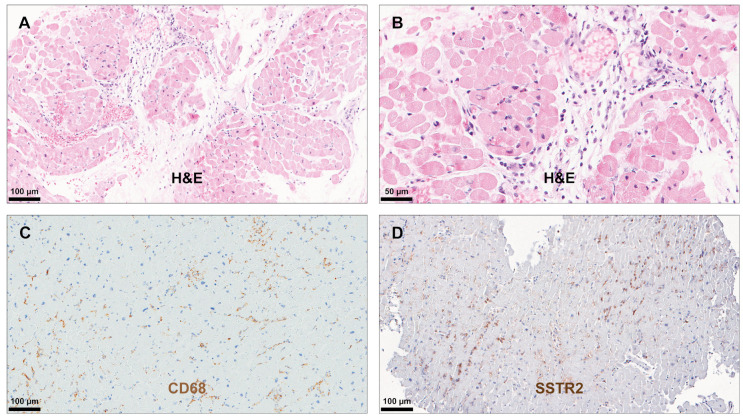
Immunohistochemical analysis in lymphocytic myocarditis. Haematoxylin and eosin (H&E) staining demonstrated infiltration of the myocardium by mononuclear inflammatory cells including lymphocytes and macrophages (**A**,**B**). Immunohistochemical staining for CD68 confirmed the presence of numerous macrophages in the myocardium (**C**), and many of the mononuclear cells showed positive staining for somatostatin receptor subtype 2 SSTR2 (**D**). CD, cluster of differentiation.

**Figure 2:**
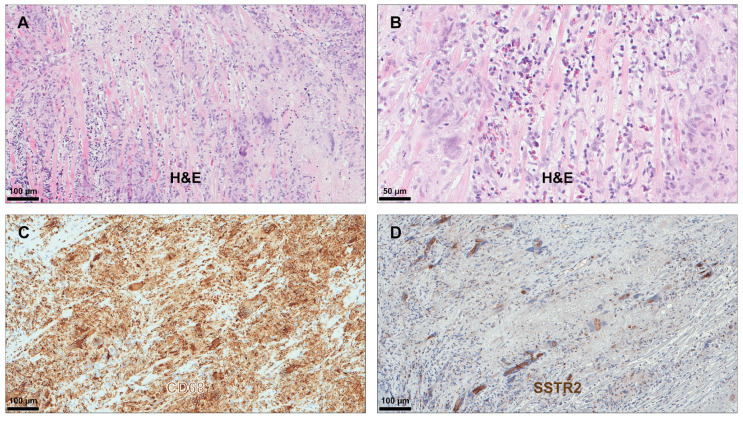
Immunohistochemical analysis in giant-cell myocarditis. H&E staining revealed intense signs of inflammation with infiltration of the myocardium by multinucleated giant cells, lymphocytes, and numerous eosinophilic leukocytes (**A**,**B**). Macrophages including multinucleated giant cells (emerging due to fusion of macrophages) showed prominent staining for CD68 (**C**) and SSTR2 (**D**). Abbreviations as in Figure 1.

**Figure 3:**
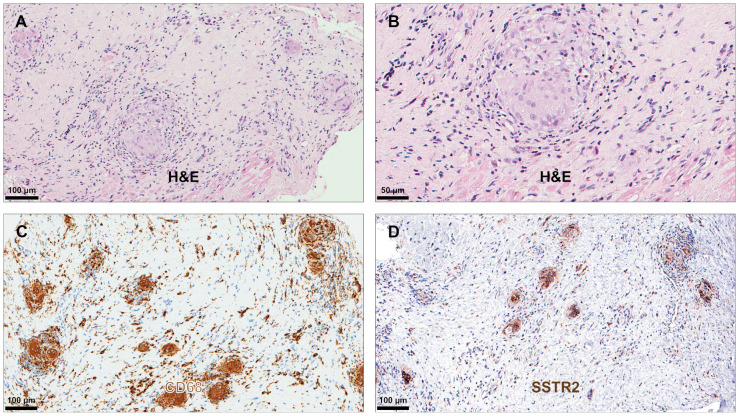
Immunohistochemical analysis in cardiac sarcoidosis. H&E staining demonstrated a well-demarcated noncaseating granuloma with centrally located macrophages, many of which were multinucleated giant cells and surrounded by lymphocytes (**A**,**B**). Immunohistochemical staining for CD68 showed a focal granulomatous pattern with prominent staining of the multinuclear giant cells and mononuclear macrophages (**C**). Immunohistochemical staining for SSTR2 showed prominent staining of multinucleated giant cells and some mononuclear cells (**D**). Abbreviations as in Figure 1 and Figure 2.

**Figure 4:**
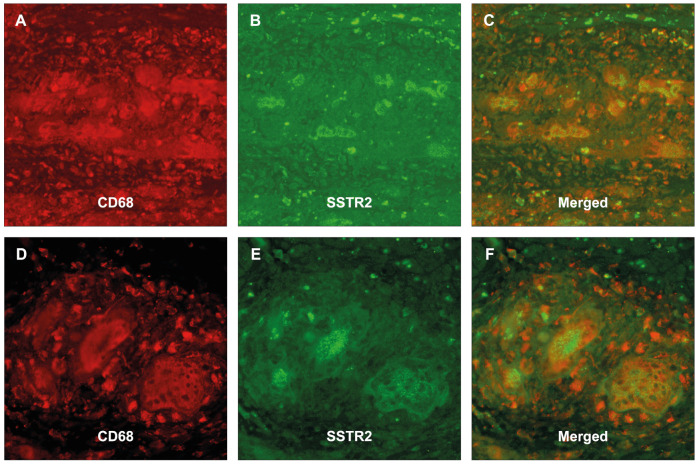
Immunofluorescence analysis in giant-cell myocarditis (**A**–**C**) and cardiac sarcoidosis (**D**–**F**). Immunofluorescence microscopy of the same section with staining for CD68 (**A**,**D**) and SSTR2 (**B**,**E**) confirmed in the merged image, (**C**,**F**) the expression of SSTR2 on CD68-positive macrophages including multinucleated giant cells (colocalization). Abbreviations as in Figure 1, Figure 2 and Figure 3.

All included patients fulfilled the histological diagnostic criteria for LM, GCM, or CS. Myocardial tissue samples from all patients revealed positive IHC staining for SSTR2 in the presence of both infiltrating mononuclear cells and multinucleated giant cells (Table 3). However, the intensity of the staining was most prominent in the presence of multinucleated giant cells. Figure 1, Figure 2 and Figure 3 illustrate typical findings in LM, GCM, and CS. Furthermore, SSTR2 positive staining was frequently observed in areas with CD68-positive macrophages including multinucleated giant cells, which emerge due to the fusion of macrophages. IF microscopy confirmed the expression of SSTR2 on CD68-positive cells in the case of patients with GCM and CS (Figure 4). In patients with LM, this IF-based confirmation was not feasible, due to technical reasons.

**Figure 5:**
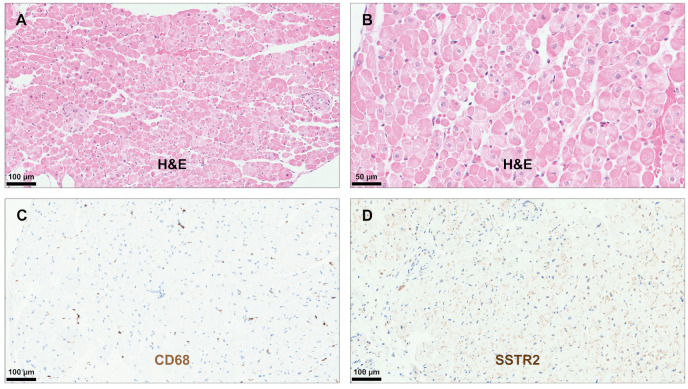
Immunohistochemical analysis in controls. H&E staining revealed a myocardium without signs of inflammatory cell infiltration and/or scars (**A**,**B**). Immunohistochemical staining for CD68 confirmed the presence of a few scattered macrophages in the myocardium (**C**) and for SSTR2 the presence of a weak, unspecific background staining (**D**). Abbreviations as in Figure 1, Figure 2, Figure 3 and Figure 4.

None of the controls showed histologic signs of myocardial inflammation and/or scars (Figure 5). Consequently, IHC staining for SSTR2 was mainly negative, due to the absence of larger inflammatory cell infiltrates. In a few control specimens, a weak positive background staining was observed, which was considered as unspecific (Figure 5).

### 3.3. Western Blot Characteristics

**Figure 6:**
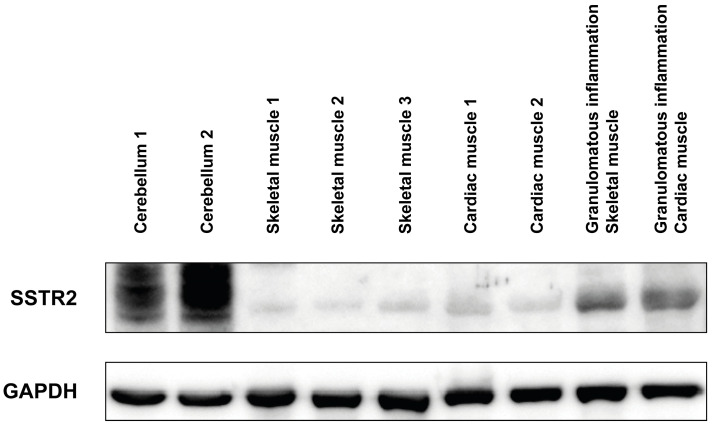
Western blot analysis. The expression of SSTR2 was upregulated in cases of granulomatous inflammation (sarcoidosis) of the skeletal and heart muscle, in contrast to the healthy skeletal and cardiac muscle. Cerebellar tissue was used as positive control. GAPDH, Glyceraldehyde-3-phosphate dehydrogenase (loading control); otherwise, abbreviations as in Figure 1, Figure 2, Figure 3, Figure 4 and Figure 5.

Western blotting confirmed the upregulated expression of SSTR2 in cases of granulomatous inflammation (sarcoidosis) of the skeletal and heart muscle, in contrast to healthy skeletal and cardiac muscle (Figure 6).

## 4. Discussion

In the present study, we systematically analysed the expression of SSTR2 on inflammatory cells in patients with biopsy- or explant-proven myocarditis. Our results show that SSTR2 is expressed at the protein level on CD68-positive macrophages and multinucleated giant cells in LM, GCM, and CS. These findings provide a clear rationale for the diagnostic use of SSTR imaging in patients with clinically suspected myocarditis.

Somatostatin receptors, which belong to the superfamily of G-protein coupled receptors, constitute of five subtypes, and show a characteristic expression pattern throughout the body [27]. It has previously been shown that SSTR2 and SSTR3 are expressed on activated monocytes, macrophages, lymphocytes, and multinucleated giant cells [10,17,28,29]. However, their role within the immune system is still not fully understood, in contrast to their well-described role in regulating gastrointestinal processes [27]. The expression of SSTRs on inflammatory cells has successfully been exploited by a novel imaging technique called SSTR imaging, using, for instance, PET/CT technology [9,30]. The feasibility of this method in patients with myocarditis has previously been shown in small studies and case reports [11,12,13,14,15,16,17,18,19,20,21,22,23]. However, little is known about the histopathological correlate of the method in patients with myocarditis. Bravo et al. described histopathologic correlations with positive IHC staining for SSTR2 in three patients with CS [15]. In case of GCM, the evidence is even more scarce and based solely on one case report [17]. No previous results are known with respect to LM. Consequently, the present study is, to the best of our knowledge, the largest available investigation concerning the expression of SSTR2 on inflammatory cells in different forms of myocarditis using histopathological techniques and Western blotting. Thereby, our study provides information concerning the expression of SSTR2 at the protein level. However, further validation of our findings is needed to provide a more complete rationale for SSTR imaging in myocarditis, namely by using, for instance, gene expression analysis (mRNA sequencing, etc.) and/or autoradiography, to confirm specific tracer binding in tissue samples.

Interestingly, the most prominent IHC staining for SSTR2 was, in the current study, found in the presence of multinucleated giant cells, which are the hallmark of GCM, but can also be found in noncaseating granulomas of patients with CS. The diagnostic relevance of these findings is still speculative, but might indicate that the obtained signal intensity, in conjunction with SSTR imaging, might be higher in cases of GCM and CS than in LM. Nonetheless, it should be kept in mind that the extent of SSTR2 staining and, thereby, the signal intensity, is also determined by the degree of myocardial inflammation itself, namely the number of inflammatory cells present within the myocardium. Future imaging studies will certainly shed further light on this aspect.

Finally, it is noteworthy that SSTRs are expressed in the healthy myocardium, although solely in the form of subtype 5 [27]. In a few of our controls, IHC staining for SSTR2 showed weak background staining without any specific link to certain cell types and/or the presence of myocardial inflammation and/or scars. Consequently, this weak background staining, which can frequently be found when using IHC staining techniques, was considered as unspecific. These findings are in line with previous observations by Bravo et al. [15]. However, recent findings indicate that SSTR subtype 1 and SSTR2 might be upregulated in areas with myocardial fibrosis [31]. However, it is still unclear whether this is due to the presence of macrophages in the fibrotic areas or due to alterations in the myocyte expression of certain SSTR subtypes. This highlights once more the need for further research to improve our knowledge on the differential expression of SSTRs on different cell types in myocarditis, including not only inflammatory cells, but also other cell types, such as fibroblast and myocytes.

Our retrospective histopathologic study has, despite its strengths, also some limitations. Firstly, the relatively small number of available patients with LM is a clear limitation of the study, as it confines the generalizability of our findings. This is, however, owed to the clinical routine at our institution, which uses mainly cardiovascular magnetic resonance imaging in these patients as the primary diagnostic tool, and only rarely an endomyocardial biopsy. Consequently, future studies with a larger sample size and even broader spectrum of underlying aetiologies are needed to confirm our findings. Secondly, it would have been of additional interest to study even the expression of SSTR3 on inflammatory cells in different forms of myocarditis. Clearly, this would have contributed to a more complete picture, as it is postulated that SSTR3 is expressed on lymphocytes in contrast to SSTR2, which is expressed on CD68-positive macrophages and multinucleated giant cells (as in the present study) [28]. However, the expression of SSTR3 on inflammatory cells is beyond the scope of the present study and remains to be explored by future investigations.

## 5. Conclusions

Our results indicate that SSTR2 is expressed on the protein level on CD68-positive macrophages and multinucleated giant cells in different forms of myocarditis, such as LM, GCM, and CS. These findings provide a clear rationale for the diagnostic use of SSTR imaging in patients with clinically suspected myocarditis.

## Figures and Tables

**Table 1:** Demographic and clinical characteristics in patients with lymphocytic myocarditis (LM), giant-cell myocarditis (GCM), and cardiac sarcoidosis (CS).

	LM (*n* = 5)	GCM (*n* = 11)	CS (*n* = 17)	Overall *p*-Value
Demographic characteristics				
Age (years)	29 (25–67)	54 (51–67)	58 (52–62)	0.096
Sex (male)	2 (40)	5 (45)	10 (59)	0.673
BMI (kg/m^2^)	29 (24–30)	23 (21–26)	26 (20–31)	0.351
Comorbidities				
Hypertension	1 (20)	2 (18)	4 (24)	0.942
Diabetes mellitus	0	1 (9)	0	0.357
Coronary artery disease	0	0	4 (24)	0.117
Clinical presentation *				
Heart failure	2 (40)	4 (36)	12 (71)	0.161
Chest pain	3 (60)	4 (36)	2 (12)	0.074
Tachyarrhythmia	0	3 (27)	4 (24)	0.440
Bradyarrhythmia/Heart block	2 (40)	1 (9)	3 (18)	0.330
Sudden cardiac death	0	1 (9)	0	0.357
Other	0	0	1 (6)	0.616
Clinical course				
Fulminant	3 (60)	4 (36)	6 (35)	0.591

Data are presented as median (interquartile range) or number (%). * Some patients presented with multiple symptoms. Abbreviations: BMI, Body Mass Index.

**Table 2:** Basic characteristics and clinical background of controls *.

Control No.	Gender	Age (Years)	Cause of Death	Clinical Background
1	M	62	Subarachnoid haemorrhage	Atrial fibrillation, previous Maze surgery
2	M	63	Cardiac arrest	Previous mitral valve replacement
3	F	50	Intracerebral haemorrhage	Previous ventricular tachycardia, suspected previous AMI, suspected Takotsubo cardiomyopathy in the acute setting
4	F	63	Ischemic cerebral oedema, due to cardiac arrest	Ischemic heart disease, obesity, hypertension, diabetes mellitus type 2, hypothyroidism, renal insufficiency, emphysema
5	F	19	Ischemic cerebral oedema due to cardiac arrest	Anorexia
6	F	42	Intracerebral haemorrhage	Takotsubo cardiomyopathy in the acute setting
7	F	43	Ischemic cerebral oedema due to cardiac arrest caused by major bleeding	None
8	M	24	Cardiac arrest	Hypertrophic cardiomyopathy, athlete

* Multi-organ donors that were not suitable for cardiac transplantation. Abbreviations: AMI, acute myocardial infarction; F, female; M, male.

**Table 3:** Individual patient characteristics and immunohistochemical staining results for somatostatin receptor subtype 2 (SSTR2) in lymphocytic myocarditis (LM), giant-cell myocarditis (GCM), and cardiac sarcoidosis (CS).

Patient No.	Gender	Age (Years)	Diagnosis	Endomyocardial Biopsy */Explant	SSTR2 Positive Staining	
					Mononuclear Inflammatory Cells	Multinuclear Giant Cells
1	F	29	LM	2/3	+++	
2	F	71	LM	2/4	+	
3	F	21	LM	3/3	+	
4	M	29	LM	4/4	+	
5	M	63	LM	3/4	+	
6	F	71	GCM	4/4	+	+++
7	F	53	GCM	8/8	++	++
8	M	54	GCM	3/3	++	+++
9	M	78	GCM	3/3	-	++
10	M	54	GCM	4/5	++	+++
11	M	60	GCM	1/2	++	++
12	M	19	GCM	1/1	+	++
13	F	56	GCM	2/2	+++	+++
14	F	48	GCM	7/7	++	+++
15	F	51	GCM	Explant	+	++
16	F	74	GCM	2/2	++	++
17	F	54	CS	2/4	++	+++
18	M	59	CS	2/2	++	+++
19	M	63	CS	2/5	++	+++
20	M	58	CS	2/6	++	+++
21	M	52	CS	2/3	+	+++
22	M	58	CS	1/2	+	+++
23	F	52	CS	1/4	+	+++
24	M	48	CS	3/5	+	+++
25	F	51	CS	Explant	+	+++
26	F	66	CS	Explant	+	+++
27	M	61	CS	1/4	++	+++
28	M	64	CS	4/7	++	+++
29	M	63	CS	Explant	++	+++
30	F	57	CS	Explant	+	+++
31	F	44	CS	Explant	+	+++
32	F	59	CS	3/4	++	+++
33	M	55	CS	1/4	+	+++

* No. of pieces with inflammation/total number of available biopsy pieces. Grading of SSTR2 staining intensity: +++ prominent, ++ moderate, + light, and - absent. Abbreviations: F, female; M, male.

## Data Availability

The data generated in the present study are not publicly available.

## Supplementary Materials

The following supporting information can be downloaded at: https://www.mdpi.com/article/10.3390/diagnostics14212374/s1, Figure S1: Grading of immunohistochemical staining intensity of inflammatory cells for somatostatin receptor subtype 2.
