# Supplementary material for: Histopathological Evaluation of Somatostatin Receptor 2 Expression in Myocarditis—Rationale for the Diagnostic Use of Somatostatin Receptor Imaging
Source: Diagnostics (Basel). 2024 Oct 24;14(21):2374. doi: 10.3390/diagnostics14212374 (PMC11545006; doi:10.3390/diagnostics14212374)
Supplement: Supplementary file 1 [file diagnostics-14-02374-s001.zip › diagnostics-3255011-supplementary.pdf]

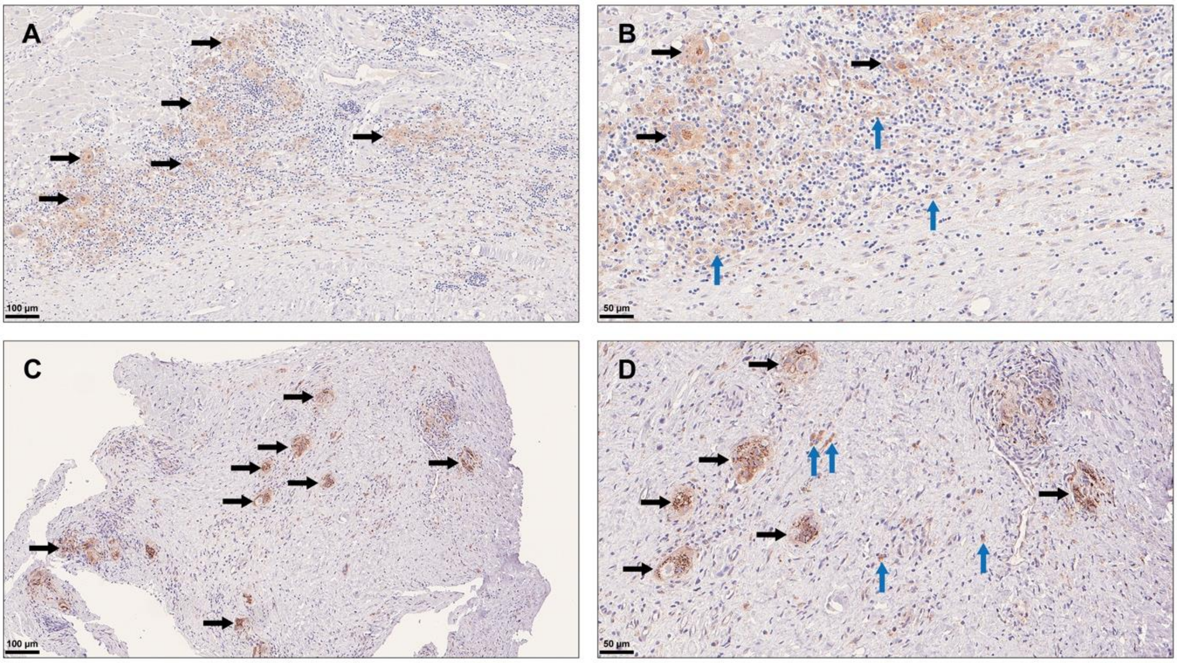

Figure S1. Grading of immunohistochemical staining intensity of inflammatory cells for somatostatin receptor subtype 2. Mononuclear inflammatory and multinucleated giant cells are indicated by blue and black arrows, respectively. In patient 15 (A and B) the mononuclear inflammatory cells were graded as light (+) and the multinuclear giant cells as moderate (++). In patient 17 (C and D) the mononuclear inflammatory cells were graded as moderate (++) and the multinucleated giant cells as prominent (+++). Detailed information concerning every patient can be found in Table 3.
